# Enzymatic testing for mucopolysaccharidosis type I in Kuwaiti newborns: a preliminary study toward newborn screening

**DOI:** 10.3389/fped.2024.1376053

**Published:** 2024-07-15

**Authors:** Hind Alsharhan, Mohammad Z. Haider, Bann Qadoura, Mariam Ayed, Gursev S. Dhaunsi, Hessa Alkandari

**Affiliations:** ^1^ Department of Pediatrics, Farwaniya Hospital, Ministry of Health, Sabah Al-Nasser, Kuwait; ^2^ Department of Pediatrics, Health Sciences Centre, College of Medicine, Kuwait University, Safat, Kuwait; ^3^ Kuwait Medical Genetics Center, Ministry of Health, Sulaibikhat, Kuwait; ^4^ Department of Pediatrics, Amiri Hospital, Ministry of Health, Kuwait City, Kuwait; ^5^ Department of Neonatology, Farwaniya Hospital, Ministry of Health, Sabah Al-Nasser, Kuwait

**Keywords:** lysosomal storage disorder, newborn screening, mucopolysaccharidosis, enzymatic testing, genetic testing

## Abstract

Mucopolysaccharidosis type I (MPS I) is an autosomal recessive lysosomal storage disorder characterized by deficient or absent *α*-L-iduronidase (IDUA) enzyme activity due to pathogenic variants in the *IDUA* gene. Early treatment with hematopoietic stem cell transplantation and/or enzyme replacement therapy is associated with improved outcomes in this progressive multisystem disease. The diagnosis is usually delayed due to late presentation and non-specific symptoms, which result in high morbidity and mortality. The incidence of MPS I is unknown in Kuwait. This pilot study was undertaken to screen MPS I in all Kuwaiti neonates born at Farwaniya Hospital (FH), a major center in Kuwait, over 12 months. This study examined the incidence of MPS I for inclusion in the national newborn screening (NBS) to enable its early detection and adequate treatment. All Kuwaiti neonates born at FH between December 2021 and December 2022 were screened for MPS I. The screening consisted of determining IDUA enzyme activity in dried blood spot-derived samples using tandem mass spectrometry. A follow-up genetic analysis of the *IDUA* gene has been planned to screen the cases with diminished IDUA enzyme activity as second-tier testing. A total of 618 newborns, including 331 (54%) boys and 287 (46%) girls, were screened. Of them, 20 had deficient IDUA enzyme activity but showed negative genetic testing. However, we have diagnosed one additional female infant with MPS I who belonged to FH, but the parents chose to deliver in a private hospital. The molecular genetic study revealed the presence of a previously reported pathogenic nonsense variant in the *IDUA* c.1882C>T, which is associated with severe phenotype. That being included, MPS I is estimated to be approximately 0.2% of all screened cases in Kuwait. Our study is the first to evaluate the incidence of MPS I in Kuwait. Given the single center, small number of screened infants, and the short study duration thus far, it is premature to calculate the incidence. It is anticipated that as the study continues, we would be able to estimate the incidence in our population correctly. Screening newborns in all maternity hospitals in Kuwait is necessary to calculate the actual incidence of this severe disorder. Still, our preliminary data support the inclusion of MPS I in national NBS program to allow early initiation of treatment and thus improve disease outcome.

## 1 Introduction

Mucopolysaccharidosis type I (MPS I, MIM #607014) is an autosomal recessive subtype of lysosomal storage disorders (LSD) caused by the build-up of glycosaminoglycans (GAGs) in the lysosomes, which occurs as a result of an absence or deficiency of *α*-L-iduronidase (IDUA) enzyme commission (EC) number 3.2. 1.76, encoded by the *IDUA* gene. The IDUA enzyme is essential for the breakdown of two GAGs, heparan sulfate and dermatan sulfate (1–3). The accumulation of lysosomal GAGs results in the disturbance of cellular homeostasis leading to progressive cellular damage, eventually resulting in a progressive debilitating multisystem disease with variable severity, involving musculoskeletal, cardiac, respiratory, and central nervous systems (CNS) with poor prognosis in untreated individuals, leading to death in the first decade (1, 4, 5). MPS I has been classified into three different forms depending on severity and age of onset: Hurler (severe), Scheie (mild), and Hurler/Scheie (intermediate), with no biochemical differences among them and a tendency to be described as having either a “severe” or “attenuated” form of MPS I (1, 6–8). Infants are typically born with no identifiable clinical symptoms, and based on the severity of the disease, they will usually be symptomatic within the first 3 years of life (1, 9). MPS I is a pan-ethnic disorder affecting boys and girls equally, with an incidence in the range of 0.54–1.84 cases per 100,000 newborns (8). The prevalence of the severe form is estimated to be 1:100,000 newborns, and that of the attenuated form, 1:500,000 (1). However, due to frequent misdiagnosis and underdiagnosis of diseases, especially the milder forms, it is difficult to determine the true frequency of this disease in the general population (1, 2). Even though it is believed that the incidence of inherited genetic disorders, in general, is higher in Arab countries compared to the other parts of the world due to the high consanguinity rates, there are no reliable data regarding the incidence and prevalence of MPS I in the Middle East and North Africa (MENA) region (10–13). Since the incidence of MPS I in Kuwait is unknown, this pilot study, whose preliminary results we here describe, was carried out exploring the possibility of including it in the national newborn screening (NBS) program, thereby aiding in the assessment of its actual incidence and facilitating an early diagnosis.

The available treatment options for MPS I include hematopoietic stem cell transplantation (HSCT) and/or enzyme replacement therapy (ERT) (laronidase; Aldurazyme® by BioMarin/Genzyme, California, USA, a human recombinant *α*-L-iduronidase, weekly intravenous infusion), which are more effective when administered before the presence of clinical manifestations (1, 6, 14–18). Many factors have stimulated the efforts to incorporate LSD in the NBS, especially MPS I, as follows: (1) the availability of therapies for MPS I and other LSD; (2) the development of new screening tests using dried blood spots (DBS); and (3) the fact that early diagnosis and initiation of treatment are known to improve the outcome (1, 9, 19–21). Like many rare disorders, the early diagnosis of MPS I is a challenge. Still, IDUA enzyme activity and GAGs accumulation can be measured at birth in DBS. Thus, these methods have been adopted by various NBS programs enabling MPS I detection before the appearance of clinical signs and symptoms (9, 19, 20). To follow those recommendations, MPS I NBS was added to the recommended uniform screening panel (RUSP) in the USA in 2016 to be included in the list of disorders that are screened in different states’ universal NBS programs (5, 22).

MPS I was initially screened with a single-tier approach using IDUA enzyme activity and resulted in extremely low positive predictive values due to the low cutoff and pseudodeficiency of the *IDUA* gene (19). However, a second-tier biomarker was needed for precision and improved positive predictive values in the USA, which included measurement of multiple GAGs in DBS using tandem mass spectrometry (LC-MS/MS) (9, 19). This two-tier screening is suggested to be the gold standard for diagnosing MPS I patients in newborns and has been implemented in the NBS program in the USA (9). It is anticipated that subsequent evaluation would include further biomarker studies, thorough clinical assessment, and molecular genetic testing.

Ultimately, we wanted to estimate the frequency of MPS I in Kuwait, while exploring the possibility of including it in the expanded national NBS program, especially because of the availability of therapeutic measures (23).

## 2 Methods

### 2.1 DBS collection protocol and screening population

All Kuwaiti neonates born at Farwaniya Hospital (FH), one of the six main government hospitals in Kuwait, between December 2021 and December 2022 were included in this study and screened for MPS I. Following the national NBS protocol that was previously described (23), 618 DBS samples were collected within 48–72 h of life but on a separate Whatman 903 filter card (GE Healthcare Ltd., New Jersey, USA) from which other NBS tests were performed. The DBS cards were then gathered, stored at room temperature, and sent to an analytical laboratory for analysis in Austria.

### 2.2 Ethical approval

This research study was approved by the Ethical Committee Board at the Ministry of Health, Kuwait as well as the Kuwait University College of Medicine's Institutional Review Board, following the declaration of Helsinki. The study number is MK02/21. Written informed consent for the research study and publication data was obtained from the parents of each newborn.

### 2.3 Analytical methods

The screening consisted of a multiplex enzyme assay, quantitating IDUA enzyme activity together with enzyme activities for iduronate-2-sulfatase (IDS, EC 3.1.613), associated with MPS II if deficient, alpha-N-acetylglucosaminidase (EC 3.2.1.50), associated with MPS III if deficient, N-acetylgalactosamine-6-sulfatase (EC 3.1.6.4), associated with MPS IVA if deficient, arylsulfatase B (EC. 3.1.6.12), associated with MPS VI if deficient, and beta-glucuronidase (EC 3.2.1.31), associated with MPS VII if deficient, in DBS by LC-MS/MS as previously described (24–29). Three punches were taken per DBS: one was submitted to the multiplex enzyme assay and the other two were held for potential genotyping. The protocol for the enzymatic assay is as follows: a 3-mm punch of DBS was incubated in a buffer containing six substrates and their respective internal standards overnight. A liquid–liquid extraction using aqueous NaCl and ethyl acetate was performed. Subsequently, the ethyl acetate layer was collected and dried. The residue was consequentially resuspended in solvent for auto-sampling for tandem mass spectrometry analysis. The cut-off value of IDUA activity was >1.5 μmol/L/h and has been based on extensive samples from the Middle East population.

Given its unavailability, blood GAG quantification was not performed as a second-tier test; instead, molecular genetic testing was performed as second-tier test, which indeed serves as a feasible and reliable alternative for identifying the condition.

### 2.4 Molecular genetic testing

A follow-up genetic analysis of the *IDUA* gene has been performed on the cases with diminished IDUA enzyme activity as second-tier testing. Genomic DNA was isolated from the same filter card used for enzymatic analysis as previously described (24, 30) using the Chemagic 360 extraction instrument and DNA isolation kit (PerkinElmer, Waltham, MA, USA). DNA was extracted in phosphate-buffered saline by incubation at 37 °C overnight. Polymerase chain reaction (PCR) amplifications and sequencing of coding exons and flanking intronic regions was performed using oligonucleotide primers. PCR products were purified and sequenced using a DNA sequencer (HiSeq 2,500 platform; Illumina, San Diego, CA, USA). DNA libraries were prepared using the TruSight One Sequencing Panel (Illumina). Variant calling was performed using the DRAGEN pipeline (Illumina). All amplified fragments flanking the exons were analyzed to identify variations (24, 30). Alignment of sequence reads to the human genome (GRCh38) was performed. MANE Select reference sequence used IDUA: CM000666.2. The genetic nomenclature refers to the Human Genome Variation Society (HGVS). The pathogenicity of any detected variants has been predicted using American College of Medical Genetics and Genomics (ACMG) standards and guidelines for the interpretation of sequence variants (31).

## 3 Results

**Figure 1:**
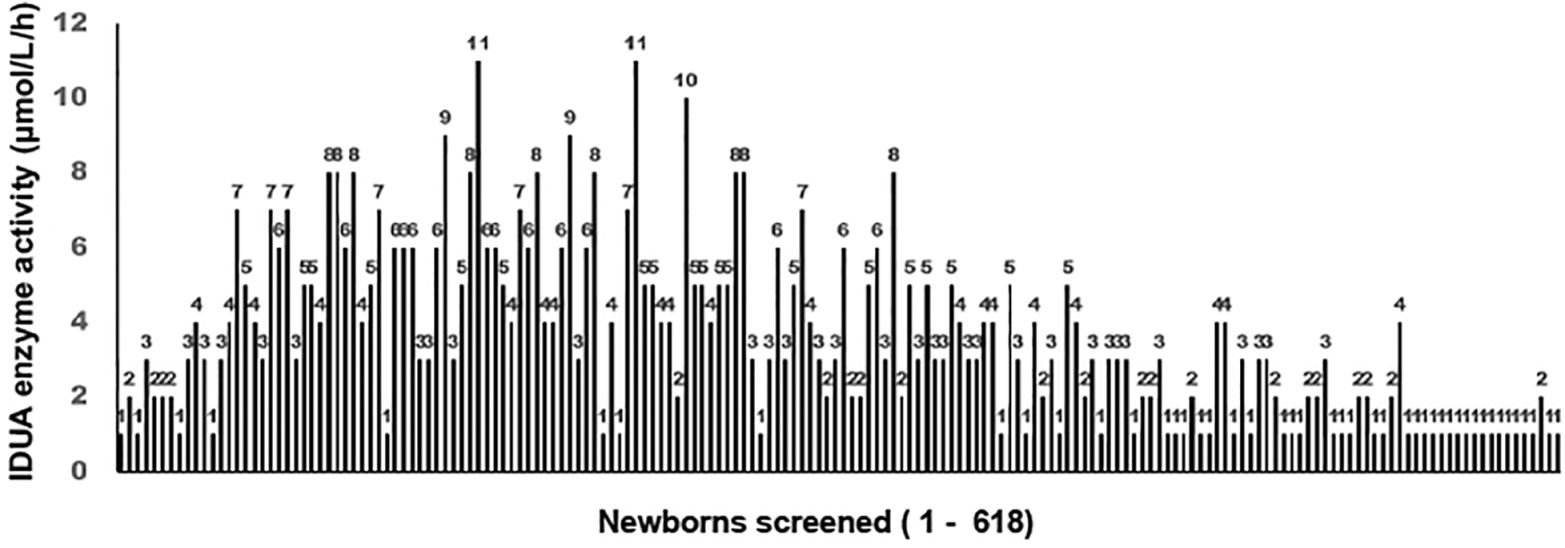
Enzyme activity (µmol/h/L) of IDUA in DBS samples of newborns (*n* = 618). IDUA activity in all samples was in the range of 0.4–11, with a median value of 6. The mean IDUA activity was 7.8 ± 5.1.

A total of 618 newborns, including 331 (54%) boys and 287 (46%) girls, were screened between December 2021 and December 2022. All the newborns were Kuwaiti descendants. Of them, 20 (9 boys, 11 girls) had deficient IDUA enzyme activity ≤1.5 μmol/L/h, in the range of 0.4–1.5 μmol/L/h. However, all had negative genetic testing for the *IDUA* gene, resulting in a false-positive rate of 3.2% (Table 1 and Figure 1). None of these newborns were heterozygous for variants in the *IDUA* gene. In addition, we diagnosed one female infant with MPS I, who belonged to the FH residential area, but whose parents had chosen to deliver in a private hospital instead of FH and thus was not screened at birth. However, she was diagnosed with MPS I at 3 months of age. That being included, MPS I is estimated to be approximately 0.2% of all screened cases in Kuwait, which is a relatively high incidence rate compared to the rest of the world. The time for DBS analysis has ranged from 1 week to 10 week 2 days due to delays in shipping the samples to the laboratory for analysis. The turnaround time for the genetic testing for final confirmation was in the range of 2–11 weeks (Table 1). We detected three newborns with reduced IDS enzyme activity (EC 3.1.613) associated with MPS II (Hunter syndrome, MIM #309900), with only one of them confirmed to be affected based on molecular genetic testing (Table 2). In addition, another newborn had borderline N-acetylgalactosamine-6-sulfate sulfatase enzyme activity (EC 3.1.6.4) at 0.2 (cut-off >0.2 μmol/L/h). Decreased or absent GALNS activity is associated with MPS IVA (Morquio syndrome; MIM #253000). Thus, a second-tier genetic test was carried out on this neonate's sample, with no pathogenic variants detected in the *GALNS* gene. The delay in the processing of the sample was 9 weeks 6 days.

**Table 1:** Newborns with false-positive reduced IDUA enzyme activity.

Newborn	Sex	Delay in analysis	IDUA enzyme activity (cut-off > 1.5 μmol/L/h)	*IDUA* genetic testing
1	M	9 weeks 4 days	1.2	Negative
2	M	7 weeks 4 days	0.7	Negative
3	M	6 weeks 5 days	0.9	Negative
4	M	6 weeks 3 days	0.4	Negative
5	M	5 weeks 6 days	1.3	Negative
6	M	8 weeks 3 days	0.8	Negative
7	M	9 weeks 3 days	0.5	Negative
8	M	7 weeks 4 days	1.2	Negative
9	F	6 weeks 3 days	1.5	Negative
10	M	7 weeks 5 days	1.3	Negative
11	F	6 weeks 3 days	0.7	Negative
12	F	7 weeks 1 days	0.7	Negative
13	F	10 weeks 2 days	1.3	Negative
14	F	7 weeks	1.0	Negative
15	F	7 weeks 3 days	0.8	Negative
16	F	6 weeks 1 day	0.9	Negative
17	F	9 weeks 4 days	1.1	Negative
18	F	9 weeks 2 days	0.5	Negative
19	F	8 weeks	1.0	Negative
20	F	8 weeks 3 days	0.6	Negative

F, female; M, male.

**Table 2:** Newborns with reduced IDS enzyme activity.

Newborn	Sex	Delay in analysis	IDS enzyme activity (cut-off >2.5 μmol/L/h)	*IDS* genetic testing
1	M	1 weeks 0 days	2.3	Likely benign hemizygous variant c.641C>T; p.(Thr214Met)
2	F	1 weeks 3 days	2.4	Negative
3	M	7 weeks 2 days	1.9	Likely pathogenic hemizygous variant c.1036G>A; p.(Ala346Thr)

F, female; M, male.

### 3.1 Detailed clinical and diagnostic information of the confirmed positive case

The single confirmed case of MPS I presented at the age of 3 months 15 days and was diagnosed at that time. The delay in diagnosis was mainly due to the delivery of the infant in a private hospital rather than at FH, despite belonging to the FH residential area. The diagnosis was confirmed via both biochemical and genetic testing; enzymatic testing showed reduced IDUA activity at 0.1 μmol/L/h (cut-off >1.5) and genetic testing revealed a previously reported pathogenic homozygous nonsense variant in the *IDUA* gene (c.1882C>T; p.Arg628Ter). She was born full term with no antenatal complications to consanguineous parents. Her clinical examination was notable for facial coarse features, macrocephaly (head circumference >90th percentile), and extensive Mongolian macules over the back, lower, and upper extremities. Imaging was remarkable for mild hepatosplenomegaly and dysostosis multiplex; echocardiogram showed mildly thickened anterior mitral leaflet and aortic valve. She was noticed to have extensive Mongolian macules, noisy breathing, snoring, and recurrent upper respiratory tract infections since the age of 1 month, requiring courses of oral antibiotics, inhaled and systemic steroids, and inhaled bronchodilators, prescribed by a pediatrician and pediatric pulmonologist. She was started on a weekly ERT (laronidase) infusion at 4 months of age and underwent successful HSCT at the age of 9 months. She is currently 18 months old, doing well, and developing adequately.

## 4 Discussion

Newborn screening in Kuwait was expanded in 2014 to include 22 endocrine and metabolic disorders with the goal of detecting and treating certain medical conditions early to improve the outcome in a cost-effective manner (23). MPS I is currently not included in the Kuwait national NBS program. Disease prevalence and the frequency of carriers in the Kuwaiti population are unknown. We conducted this pilot study at FH, one of the six main government hospitals, to evaluate the incidence of the disease in Kuwait and its carrier frequency, as a first step toward including it in the national NBS program.

Kuwait is a small country located in the Arabian Peninsula, with a population of 4.45 million people, of which 1.45 million are Kuwaiti citizens. The vast majority of healthcare services in Kuwait is distributed among 6 main governmental general hospitals and 16 private hospitals. Despite the free healthcare services provided by the government hospitals, Kuwaiti citizens prefer seeking medical management through the private medical center to reduce the wait time and for the convenience of scheduled appointments (32). All Kuwaiti citizens and foreigners have a mandatory Kuwait civil identification card (ID) that allows access to certain governmental services including the government hospitals. Therefore, each of the six government hospitals is assigned to a certain residential area, which is documented in the ID of all individuals living in Kuwait.

In this study, we screened a total of 618 Kuwaiti newborns delivered at FH for reduced IDUA enzyme activity using DBS. In Kuwait, during 2021–2022, there were a total of 33,229 live births among Kuwaiti nationals. This means that our cohort accounts for approximately 1.86% of all Kuwaiti live births. As a second-tier test, we performed DNA sequencing of the *IDUA* gene instead of relying solely on glycosaminoglycans for confirmation, which indeed serves as a feasible and reliable alternative for identifying the condition.

While the number of newborns screened may seem small, it is important to consider the context of the total Kuwaiti population. We identified 20 neonates with reduced IDUA enzyme activity but negative for biallelic pathogenic variants in the *IDUA* gene as second-tier testing. None of them were carriers for the disease and, unfortunately, the total carrier frequency of the *IDUA* variant could not be measured in Kuwaiti newborns.

The reduced IDUA enzyme activity in the 20 cases resulted in a false-positive rate of 3.2% (Table 1) and could be attributed to several factors: (1) the prolonged transfer time to the laboratory for analysis and thus exposure to the heat; (2) pseudodeficiency; or (3) a heterozygous carrier for a pathogenic variant for the *IDUA* gene. However, none of these cases were heterozygous for any pathogenic variant in the *IDUA* gene.

We identified one affected female infant with MPS I at 3 months of age within the study period who belonged to the study site (FH) but was not originally recruited into the study as she was born at a private hospital, leading to the delay in diagnosis. Her diagnosis was established based on reduced IDUA activity and positive genetic testing for a homozygous pathogenic nonsense variant in the *IDUA* gene (c.1882C>T; p.Arg628Ter), resulting in an incidence of 0.2%. This variant has been associated with a severe phenotype of MPS I (Hurler syndrome). It has been frequently reported in homozygous form in affected Kuwaiti individuals and is considered to be a founder variant in Kuwait (33, 34). The variant (c.1882C>T; p.Arg628Ter) was identified in 40 out of 44 tested individuals with severe MPS I from Kuwait, accounting for 90% of disease-causing alleles in all individuals per our internal registry at Kuwait Medical Genetics Center (34).

This variant has been previously reported as compound heterozygous with another pathogenic variant in the *IDUA* or in the homozygous state. There is a close genotype–phenotype correlation among individuals with MPS I (1, 35). However, it is well known that the combinations of two null alleles are associated with a more severe phenotype, while at least one missense or intronic variant is associated with a milder disease due to some residual enzyme activity. Homozygosity or compound heterozygosity of this nonsense variant (p.Arg286Ter) has been consistently associated with a severe phenotype (Hurler syndrome) due to the premature protein truncation of about 5 °C-terminal residues resulting in non-functional protein (34–40). However, there are two reported individuals with MPS I: one with the same homozygous variant p.Arg286Ter and the other is compound heterozygous for p.Arg268Ter/p.Gln70Ter, both reported with an attenuated phenotype (Hurler/Scheie), even though the phenotype of the second described case is consistent with severe disease (Hurler) (41, 42).

However, when the variant is compound heterozygous with a missense variant, it has been associated with an attenuated phenotype (Hurler/Scheie) or the severe form (Hurler) (43, 44). This variant has been previously reported in individuals with MPS I of Middle Eastern origin (Turkey, Tunisia, Saudi Arabia, and Kuwait) (34, 38, 39, 44, 45).

The early diagnosis of this case has led to early initiation of treatment (ERT), at approximately 4 months of age, followed by HSCT at 9 months with a better outcome. This is the first study in Kuwait and the MENA region determining the incidence of MPS I.

Further screening of all Kuwaiti infants delivered at the other governmental and private hospitals is required to obtain a more accurate incidence of MPS I in Kuwait and facilitate the implementation of a nationwide screening program for MPS I. The results reported in this study add up to the discussion on the possibility of including MPS I in the national NBS program, given the severity of the founder mutation and the effectiveness of early management. If MPS I is to be included in the NBS in Kuwait, we recommend obtaining IDUA enzyme activity in DBS, followed by full DNA sequencing of the *IDUA* gene as second-tier testing. This would not only help in achieving a complete genotype of the *IDUA* in our population but may also confirm that the nonsense variant (c.1882C>T; p.Arg628Ter) is the most common variant in Kuwaiti population. It will also aid in estimating the carrier frequency for this variant in the Kuwaiti population. Furthermore, it would also allow for the establishment of genotype-phenotype associations, thereby improving the ability to make appropriate therapeutic decisions.

This pilot study has several limitations that should be acknowledged. First, the screening period was relatively short, spanning only 1 year. In addition, the study was conducted in a single center, FH, which led to a small sample size. This small sample size hindered the accurate calculation of disease incidence. Another limitation of the study was the prolonged shipping of DBS samples to an external laboratory. This led to both false-positive results and significant delays in turnaround time. However, it is worth noting that this limitation could be addressed in the future by establishing an in-house laboratory, which would reduce shipping time and improve result accuracy. Blood GAG is considered ideal as second-tier testing; however, its availability is limited. Owing to the small size of the pilot population and the aim to identify all cases of MPS I, the decision has been made to prioritize molecular genetics as the second-tier testing option. This approach is more feasible for a small population such as Kuwait, as developing multiple technically challenging second-tier tests with low test volumes would be impractical. Therefore, evaluating the feasibility of molecular genetic testing as the second tier is a logical step. In addition, there is potential to incorporate blood GAGs as a third-tier test or as part of the overall diagnostic evaluation process. Despite these limitations, it is worth mentioning that our study group consisted of 618 individuals. Although this may appear relatively small, it is reasonable in the context of the total Kuwaiti population. This sample size allowed us to explore the possibility of including this disorder in the country-wide neonatal screening program.

## 5 Conclusion

This is the first study in Kuwait and the MENA region to evaluate the incidence and genotype of MPS I via screening asymptomatic newborns. Although none of the 618 screened newborns were confirmed to be affected, we have identified and confirmed one case of MPS I that would have been detected if delivered at FH, resulting in an estimated incidence of MPS I of 0.2% of all screened cases in Kuwait, which is a relatively high incidence rate compared to the rest of the world. A larger cohort is required to have an accurate estimate of the incidence of MPS I in the Kuwaiti population and to enforce its incorporation into the national NBS program, as effective therapeutic measures are available.

## Data Availability

The original contributions presented in the study are included in the article/Supplementary Material, further inquiries can be directed to the corresponding author.
